# Comparative Toxicological Profiling Reveals Divergent Redox, Mitochondrial, and Cellular Toxicity to Structurally Diverse Opioids

**DOI:** 10.3390/jox16060193

**Published:** 2026-10-10

**Authors:** Alica Pizent, Antonio Zandona, Andreja Jurič, Blanka Tariba Lovaković, Dubravka Rašić, Nevenka Kopjar, Katja Vuković, Maja Katalinić, Arnes Rešić, Irena Canjuga, Ana Lucić Vrdoljak, Irena Brčić Karačonji

**Affiliations:** 1 Division of Occupational and Environmental Health, Institute for Medical Research and Occupational Health, 10000 Zagreb, Croatia; apizent@imi.hr; 2 Division of Toxicology, Institute for Medical Research and Occupational Health, 10000 Zagreb, Croatia; ajuric@imi.hr (A.J.); btariba@imi.hr (B.T.L.); rasic@imi.hr (D.R.); nkopjar@imi.hr (N.K.); kvukovic@imi.hr (K.V.); mkatalinic@imi.hr (M.K.); alucic@imi.hr (A.L.V.); ibrcic@imi.hr (I.B.K.); 3 Department of Pediatric Clinical Pharmacology and Toxicology, Children’s Hospital Zagreb, 10000 Zagreb, Croatia; aresic@fzz.unist.hr; 4 Department of Health Studies, University of Split, 21000 Split, Croatia; 5 Department of Nursing, University North, 42000 Varaždin, Croatia; irena.canjuga@unin.hr; 6 Faculty of Health Studies, University of Rijeka, 51000 Rijeka, Croatia

**Keywords:** drug abuse, oxidative stress, antioxidative defense, cell viability

## Abstract

Opioid neurotoxicity is increasingly associated with oxidative stress, mitochondrial dysfunction, and DNA damage; however, direct mechanistic comparisons among structurally diverse opioids remain scarce. We comparatively evaluated morphine, codeine, heroin, buprenorphine, and methadone in human SH-SY5Y neuroblastoma cells using a multiparametric approach encompassing cytotoxicity; redox homeostasis; mitochondrial membrane potential; LDH release; caspase-3, -8, and -9 activation; PI3K/Akt signaling; DNA strand breaks; and in silico pharmacokinetic profiling. The opioids exhibited clearly divergent toxicity patterns. Buprenorphine showed the highest cytotoxicity, mitochondrial depolarization, and activation of caspase-9 and caspase-3, consistent with mitochondrial caspase-dependent apoptotic signaling. Morphine also reduced mitochondrial membrane potential and activated caspase-9, whereas heroin and codeine increased caspase-9 activity without significant mitochondrial depolarization. Methadone produced the most pronounced oxidative phenotype, with increased ROS, glutathione depletion, mitochondrial depolarization, and the only significant increase in LDH release but without significant caspase activation, indicating a distinct caspase-independent injury pattern. Akt activation increased significantly after buprenorphine and morphine exposure. Morphine and codeine induced greater DNA damage than methadone at equimolar exposure, demonstrating that oxidative burden did not parallel genotoxicity. Overall, these findings reveal compound-specific redox, mitochondrial, signaling, genotoxic, and cell-death responses and show that opioid toxicity cannot be adequately explained by oxidative stress alone.

## 1. Introduction

**Figure 1:**
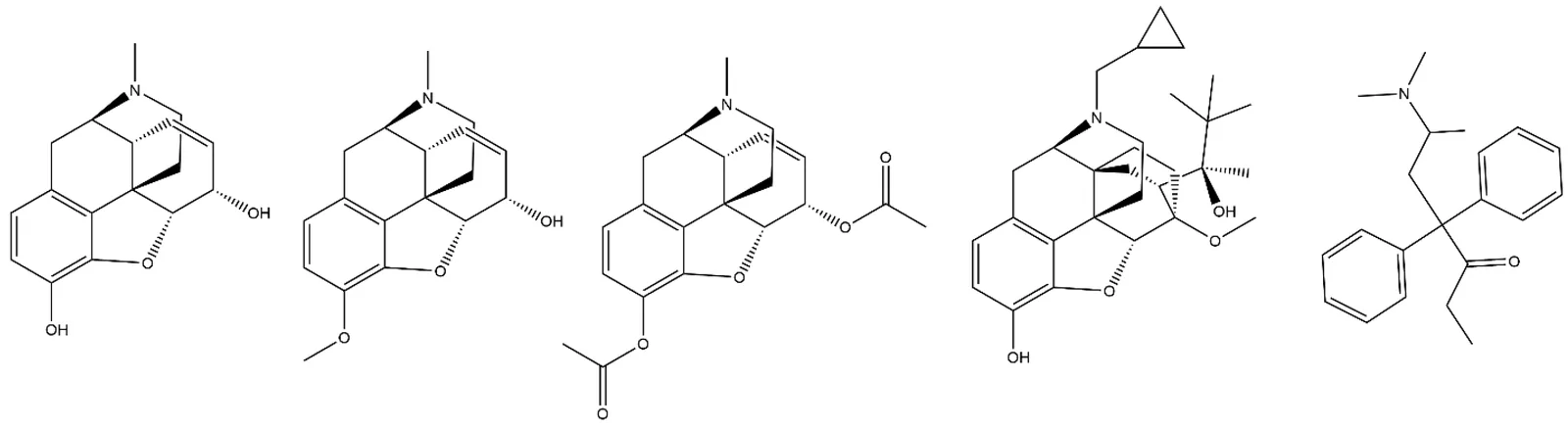
Structures of the opioids used in this study (from left to right: morphine, codeine, heroin, buprenorphine, and methadone).

Opioid compounds are a diverse group of medications essential in clinical practice for the treatment of acute and chronic pain, anaesthesia, and opioid substitution therapy. However, their use is associated with serious adverse effects, including tolerance, dependence, and neurotoxicity. The global opioid crisis has further intensified the need to understand the cellular consequences of both therapeutic and illicit opioid exposure [1,2,3]. Opioids act primarily through µ-, δ-, and κ-opioid receptors, which are G-protein-coupled receptors physiologically modulated by endogenous peptides such as endorphins, enkephalins, and dynorphins [4,5]. Exogenous opioids are structurally diverse and include natural alkaloids such as morphine and codeine, semi-synthetic derivatives such as heroin and buprenorphine, and synthetic agents such as methadone [6]. Despite structural differences (Figure 1), they share pharmacophoric elements enabling opioid receptor binding and signal transduction [7].

Contemporary toxicological studies increasingly rely on in vitro neuronal models to investigate direct cellular responses to opioids without the systemic and behavioral variables present in vivo. Such models enable comparative assessment of cytotoxicity, oxidative stress, mitochondrial dysfunction, apoptosis, and DNA damage across different compounds and exposure conditions [8,9,10,11]. SH-SY5Y human neuroblastoma cells are widely used in neurotoxicity research because of their neuronal characteristics, sensitivity to oxidative stress, and suitability for studying drug-induced cellular injury [12,13]. This model has also been applied to studies of opioid-related cellular effects [14,15,16,17].

Oxidative stress is frequently implicated in opioid-induced neurotoxicity, but the magnitude of oxidative imbalance does not necessarily predict the extent or type of cellular injury. Increased reactive oxygen species (ROS) formation, glutathione (GSH) depletion, altered antioxidant enzyme activity, and DNA damage have all been reported following opioid exposure, yet comparative studies across structurally distinct opioids remain limited. Mitochondria represent a key intersection between oxidative stress and cell-death signaling. Loss of mitochondrial membrane potential (MMP) can promote intrinsic apoptotic signaling through cytochrome c release and activation of caspase-9 and downstream executioner caspases. In SH-SY5Y cells, morphine has been shown to induce ROS-dependent, mitochondria-mediated apoptosis involving caspase-9 and caspase-3 activation [18]. In contrast, methadone has been associated with mitochondrial dysfunction and predominantly caspase-independent, necrotic-like cell death, indicating that different opioids may engage distinct mitochondrial injury pathways [19].

Opioid receptor signaling may also modulate cellular survival through the PI3K/Akt pathway. Activation of µ-opioid receptors in SH-SY5Y cells has been shown to stimulate Akt phosphorylation through PI3K and support cell survival under stress conditions [20]. Thus, early Akt activation may provide additional insight into whether opioids trigger adaptive survival signaling before later mitochondrial dysfunction and cell death.

This study aimed to compare the toxicological profiles of natural opioids morphine and codeine, semi-synthetic opioids heroin and buprenorphine, and synthetic opioid methadone in SH-SY5Y cells. We hypothesized that structurally diverse opioids would produce distinct patterns of oxidative stress, mitochondrial dysfunction, DNA damage, and cell-death signaling rather than a uniform toxicity response. Cytotoxicity, ROS generation, antioxidant enzyme activity, glutathione status, lipid peroxidation, and DNA strand breaks were evaluated together with mitochondrial membrane potential, lactate dehydrogenase (LDH) release, and activation of caspase-3, -8, and -9. PI3K/Akt activation was additionally assessed as an early survival-related signaling response. Finally, SwissADME BOILED-Egg analysis was used to provide complementary context on predicted human intestinal absorption, blood–brain barrier (BBB) permeation, and P-glycoprotein substrate status [21]. The concentrations used in the present study were selected on the basis of SH-SY5Y cell viability and were intended for comparative in vitro assessment only; they should not be interpreted as directly equivalent to clinical or subclinical in vivo exposure levels, which are additionally shaped by absorption, metabolism, protein binding, tissue distribution, and BBB transport. By integrating redox, genotoxic, mitochondrial, and cell-death endpoints within a common experimental framework, the study sought to identify shared and compound-specific mechanisms underlying opioid-induced cellular toxicity.

## 2. Materials and Methods

### 2.1. Chemicals and Cells

Analytical standards buprenorphine, codeine, methadone and morphine (each as 1 mg/mL in methanol), as well as heroin (as 0.1 mg/mL in methanol), were purchased from Lipomed (Arlesheim, Switzerland). Other reagents and chemicals used for experiments were bought from Sigma-Aldrich (Steinheim, Germany) unless otherwise stated. Hydrogen peroxide (H_2_O_2_) was purchased from Gram-mol (Zagreb, Croatia), and methanol from Kemika (Zagreb, Croatia). Staurosporine (SS) was purchased from Abcam (Trumpington, UK). The oxime 3-bis(4-chlorobenzyl)-2-[(hydroxyimino)methyl]-imidazolium bromide (VII) was a gift from Professor Ines Primožič (University of Zagreb Faculty of Science, Department of Chemistry, Zagreb, Croatia).

Cells were maintained in Dulbecco’s Modified Eagle’s Medium/F12 supplemented with 15% (*v*/*v*) fetal bovine serum, 2 mM glutamine, 1% (*v*/*v*) penicillin/streptomycin, and 1% (*v*/*v*) non-essential amino acids. All culture media and supplements were purchased from Sigma-Aldrich (Steinheim, Germany). Cells were incubated at 37 °C in a humidified atmosphere containing 5% CO_2_, with the culture medium replaced every few days. Cells were passaged according to the manufacturer’s recommendations.

The cells for the viability assay were seeded in transparent 96-well plates at a density of 20,000 cells/well one day before the experiment. For ROS, GSH, LDH and MMP measurements, cells were seeded in black 96-well plates at a density of 10,000 cells/well, while for caspase activity measurement at a density of 20,000 cells/well. For other assays (alkaline comet, malondialdehyde (MDA) and antioxidative enzymes), they were seeded in transparent 24-well plates at a density of 50,000 cells/well. For the PI3K/Akt assay, cells were seeded in transparent 12-well plates at a density of 100,000 cells/well.

The sources of human acetylcholinesterase (AChE) and butyrylcholinesterase (BChE) were native human erythrocytes and plasma, respectively, isolated from one donor’s blood (upon approvals of the Ethics Committee of the Institute for Medical Research and Occupational Health, Zagreb, Croatia Nos. 01–18/23–02-2/1 and 100–21/23–16) following a standard procedure reported elsewhere [22].

The selected range of the opioid concentrations for testing was conducted in accordance with data reported in the literature [23,24,25,26,27,28], guided by clinically relevant opioid blood concentration ranges consistent with values described in cases of non-fatal intoxication (0.4–100 µM for morphine, codeine, buprenorphine, and methadone; 0.04–10 µM for heroin). It was also taken into consideration that the total percentage of methanol (used as vehicle) in the culture medium did not exceed 3% to prevent its potential adverse effects on cell viability. Consequently, for the cell viability assay, SH-SY5Y cells were treated with the tested compounds at concentrations ranging from 0.039 to 10 µM or from 0.39 to 100 µM, depending on the original stock solutions. For all other assays, one concentration of each drug was selected according to viability results (concentration where the viability was not below 75%) as follows in Table 1. Untreated control cells were kept under the same experimental conditions. The vehicle control (methanol) in all assays did not significantly affect any of the measured endpoints (did not exceed 3%).

To ensure the reliability of the results, a minimum of three independent experiments were conducted, with each treatment performed in duplicate or triplicate.

### 2.2. Viability Assay

Cell viability and compound-induced cytotoxicity were evaluated using the CellTiter 96^®^ AQueous One Solution Cell Proliferation Assay (MTS assay; Promega, Madison, WI, USA), which reflects mitochondrial metabolic activity. The assay was performed according to a previously described protocol [29]. After 24 h of exposure, cells were washed with phosphate-buffered saline (PBS), and the MTS reagent was applied according to the manufacturer’s instructions. Absorbance was measured at 492 nm using an Infinite M200PRO plate reader (Tecan Group Ltd., Männedorf, Switzerland). Cell viability was expressed as a percentage of the untreated control and presented as mean ± standard error.

### 2.3. Selection of Concentrations Tested

The concentrations selected for subsequent experiments are presented in Table 1. They were chosen based on the cytotoxicity results obtained in the present study, with the aim of maintaining sufficient cell viability for reliable assessment of sublethal cellular effects and in accordance with established comet assay recommendations [30,31]. As these concentrations were selected according to compound-specific viability profiles, they were not equimolar and should not be interpreted as directly equivalent to clinical or systemic in vivo exposure levels.

### 2.4. Lactate Dehydrogenase (LDH) Release

Cell membrane integrity was assessed by measuring LDH release using the CytoTox-ONE™ Homogeneous Membrane Integrity Assay (Promega, Madison, WI, USA), following a previously described protocol [32]. After 24 h of treatment, cells were equilibrated to room temperature, and the assay reagent was added according to the manufacturer’s instructions. Triton X-100 (0.18%, *v*/*v*) was used as a positive control for maximal LDH release. Fluorescence was measured at 560 nm excitation and 590 nm emission using an Infinite M200PRO plate reader (Tecan Group Ltd., Switzerland). Results are expressed as percentages of maximal LDH release.

### 2.5. Oxidative Stress Parameters

#### 2.5.1. Measurement of Reactive Oxygen Species (ROS) Level

Reactive oxygen species (ROS) production was assessed using 2′,7′-dichlorodihydrofluorescein diacetate (DCFH-DA; Sigma-Aldrich, Steinheim, Germany). Cells were seeded in black 96-well plates at a density of 10^4^ cells/mL. On the day of the experiment, the culture medium was removed, cells were washed with PBS, and incubated with 20 µM DCFH-DA for 30 min at 37 °C [15]. After removal of the dye and an additional PBS wash, cells were exposed to the selected concentrations of the tested compounds (Table 1) for 24 h. Fluorescence was measured at 488 nm excitation and 525 nm emission using a Victor3™ microplate reader (PerkinElmer, Waltham, MA, USA). All measurements were performed in triplicate, and the results are expressed as percentage (%) of negative control (untreated cells).

#### 2.5.2. Measurement of Glutathione (GSH) Levels

Intracellular glutathione (GSH) levels were assessed using the fluorogenic monochlorobimane (mBCl) probe, which reacts with reduced intracellular thiols, with the resulting fluorescence predominantly reflecting cellular GSH content [33]. Cells were seeded in black, clear-bottom 96-well plates and treated in triplicate with the selected opioid concentrations listed in Table 1 for 24 h at 37 °C. After treatment, cells were washed with PBS and incubated with 20 µM mBCl in PBS for 30 min at 37 °C. Fluorescence was measured at 355 nm excitation and 460 nm emission using a Victor3™ multilabel plate reader (PerkinElmer) [15]. The obtained fluorescence arbitrary units are expressed as percentages of control (untreated cells) values.

#### 2.5.3. Measurement of Activity of Antioxidant Enzymes

Superoxide dismutase (SOD), glutathione peroxidase (GPx), and catalase (CAT) activities were determined spectrophotometrically using commercially available assay kits (Cayman Chemical Company, Tallinn, Estonia) according to the manufacturer’s instructions. After 24 h exposure to the selected opioid concentrations, cells were washed with PBS, harvested, and centrifuged at 2000× *g* for 10 min at 4 °C. The resulting cell lysate supernatants were stored at −80 °C until analysis [14]. SOD activity was measured at 505 nm and expressed as U/g protein, GPx activity at 340 nm and expressed as nmol/min/g protein, and CAT activity at 540 nm and expressed as nmol/min/g protein. Absorbance was recorded using a SpectraMax i3x microplate reader (Molecular Devices, San Jose, CA, USA). Total protein concentration in the cell lysates was determined using the Bradford method [34].

#### 2.5.4. Measurement of Malondialdehyde (MDA) Level

Malondialdehyde (MDA) levels were determined in cell lysates according to the method of Khoschsorur et al. [35] as previously described in detail [14]. After 24 h treatment, cells were washed twice with Krebs–Henseleit buffer, centrifuged at 248× *g* for 5 min, and sonicated on ice. MDA was derivatized with thiobarbituric acid following incubation at 100 °C for 30 min. Samples were analyzed by HPLC with UV detection (Shimadzu Corporation, Kyoto, Japan) using a reverse-phase C18 column. The results are expressed as percentage (%) of negative control (untreated cells).

### 2.6. Mitochondrial Membrane Potential Change

Mitochondrial membrane potential (MMP) was assessed using tetramethylrhodamine ethyl ester perchlorate (TMRE; Cell Signaling Technology Europe, Leiden, The Netherlands), following a previously described protocol [32]. After 24 h of treatment, cells were incubated with TMRE for 20 min at 37 °C and washed with PBS, and fluorescence was measured at 550 nm excitation and 580 nm emission using an Infinite M200PRO plate reader (Tecan Austria GmbH, Salzburg, Austria). The oxime VII (100 μM), previously shown to affect MMP [32], was used as a positive control. Results were normalized to untreated control cells.

### 2.7. Caspase Activity Measurement

Caspase-3, -8, and -9 activities were simultaneously assessed using the Caspase-3, Caspase-8, and Caspase-9 Multiplex Activity Assay Kit (Abcam, Cambridge, UK), following a previously described protocol [32]. After 24 h of treatment, cells were incubated with the caspase assay loading solution for 30–60 min at room temperature in the dark, according to the manufacturer’s instructions. Staurosporine (3 μM) was used as a positive control. Results were calculated according to the manufacturer’s protocol and expressed relative to untreated control cells.

### 2.8. Alkaline Comet Assay

To establish the extent of DNA strand breaks in control and treated SH-SY5Y cells, standard procedure and the Consensus Statement for the Minimum Information for Reporting Comet Assay [36] were followed. Detailed methodological descriptions have been reported in our preceding papers [14,17]. The experiment was performed in triplicate, and altogether, 300 comets for each point were measured using the Comet Assay IV^TM^ software version 4.3 (Instem-Perceptive Instruments Ltd., Stone, UK) at 200× magnification of an epifluorescence microscope (Olympus BX50, Olympus/Evident Corporation, Tokyo, Japan). Tail intensity (DNA% in the comet tail) and tail length (expressed in µm) were used as main descriptors of DNA strand breaks.

### 2.9. PI3K/Akt

PI3K/Akt pathway activation was assessed by simultaneous detection of total Akt and phosphorylated Akt (p-Akt) using the Muse™ PI3K Activation Dual Detection Assay Kit (Merck, Darmstadt, Germany). SH-SY5Y cells were seeded at a density of 100,000 cells/well in 12-well plates and treated at approximately 80–90% confluence. Following treatment, cells were washed with PBS, detached with trypsin, and collected in complete culture medium. Cell suspensions were centrifuged at 300× *g* for 5 min at 4 °C and the pellets were resuspended in 1× assay buffer. Cells were fixed by adding an equal volume of fixation buffer and incubating on ice for 10 min. After centrifugation, the pellets were resuspended in ice-cold permeabilization buffer and incubated on ice for 10 min. Following an additional centrifugation step, cells were resuspended in assay buffer and incubated with a 1:1 mixture of anti-Akt and anti-phospho-Akt antibodies for 30 min at room temperature in the dark. Cells were then washed with assay buffer, centrifuged, and finally resuspended in 200 μL of assay buffer. Samples were analyzed using a Muse™ Cell Analyzer (Merck, Darmstadt, Germany), and PI3K/Akt activation was expressed according to the relative distribution of Akt- and p-Akt-positive cell populations.

### 2.10. Human Serum Albumin Binding Screening

Binding of the tested opioids to human serum albumin (HSA) was assessed by monitoring changes in the intrinsic fluorescence of HSA. HSA was prepared at a final concentration of 1 µM in 1× PBS and incubated with each opioid at the concentration previously selected based on the cell viability results (Table 1). Fluorescence emission spectra were recorded using a Cary Eclipse Fluorescence Spectrophotometer (Agilent Technologies, Santa Clara, CA, USA) following excitation at 280 nm, over an emission range of 300–500 nm. For each measurement, 500 µL of sample was transferred to a quartz cuvette. The fluorescence intensity at 350 nm was compared with that of HSA alone and used to calculate the percentage of fluorescence quenching. Measurements of PBS and corresponding opioid solutions prepared without HSA were performed in parallel to account for background signal and possible compound autofluorescence. Changes in HSA fluorescence were used as an indicator of opioid–albumin interaction.

### 2.11. Cholinesterase Inhibition Screening

Acetylcholinesterase (AChE) and butyrylcholinesterase (BChE) inhibition was assessed using a previously described Ellman-based protocol [22,37]. The assay was performed in 96-well plates using an Infinite M200PRO plate reader (Tecan Austria GmbH, Salzburg, Austria) at 25 °C. Each opioid was tested at the concentration previously selected based on cell viability results (Table 1), while acetylthiocholine (ATCh) was applied over a concentration range of 0.1–0.5 mM. The reaction mixture contained sodium phosphate buffer (0.1 M, pH 7.4), AChE or BChE, 5,5′-dithiobis-(2-nitrobenzoic acid) (DTNB; 0.3 mM), and the tested opioid. ATCh was added last to initiate substrate hydrolysis. Enzyme activity was monitored at 436 nm for AChE and 412 nm for BChE and expressed relative to the corresponding uninhibited control.

### 2.12. In Silico Predictions

The SwissADME web tool [21] was used to predict passive human intestinal absorption (HIA), Blood–Brain Barrier (BBB) permeation, and P-glycoprotein (P-gp) substrate status of the tested opioids using the BOILED-Egg model. The model classifies compounds according to their lipophilicity (WLOGP) and topological polar surface area (TPSA), with the egg white representing a high probability of passive intestinal absorption and the yolk representing a high probability of BBB permeation [38,39]. P-gp substrate status was additionally indicated within the BOILED-Egg representation.

### 2.13. Statistics

Results obtained by the MTS, LDH, mitochondial membrane potential and capsase assays are presented as a mean with standard error (SEM) or standard deviation (SD) of at least two independent experiments in duplicate or triplicate. Statistical analysis was performed using ordinary one-way analysis of variance (ANOVA) followed by Dunnett’s multiple-comparisons test using Prism, version 9 (Graph Pad, MA, USA). Statistical significance was determined at *p* < 0.05. Data are presented as box-and-violin plots. Statistical significance of the results regarding oxidative stress biomarkers was evaluated using ANOVA with post-hoc Tukey HSD test (Statistica software—Data Science Workbench, version 14. (License No. 14.0.0.15; TIBCO Software Inc. 2020; Palo Alto, CA, USA)). The same software was used to determine the statistical significance of measurement data obtained by the alkaline comet assay. They were initially analyzed using descriptive statistics, calculating mean, median, standard deviation, standard error, and range of measured values (min.-max.) for tail intensity and tail length in each experimental group. To assess inter-group statistical significance, data were subjected to one-way analysis of variance (ANOVA) followed by Tukey’s HSD post-hoc test, after applying a logarithmic transformation to achieve a normal distribution. The results were displayed graphically using a Box-Whisker chart. Statistical significance was determined at *p* < 0.05.

## 3. Results

### 3.1. Viability

**Figure 2:**
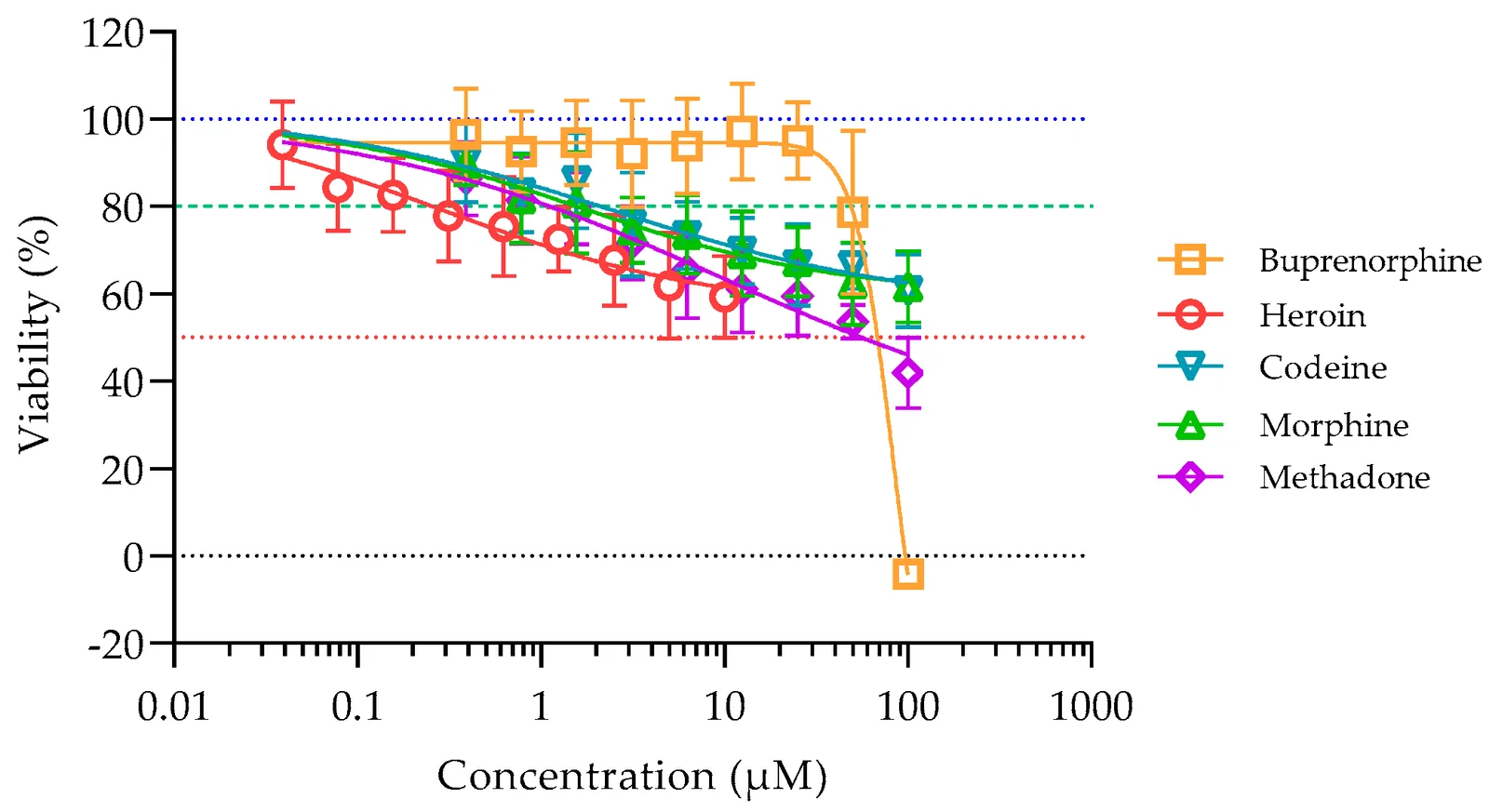
Viability of human neuroblastoma SH-SY5Y cells exposed to buprenorphine, codeine, morphine, methadone (0.39–100 μM), and heroin (0.039–10 μM) for 24 h expressed as percentage relative to control untreated cells. Legend: Dotted black line—0% viable cells; dotted red line—50% viable cells; green dashed line—80% viable cells (minimum of concentrations used for detailed experiments); dotted blue line—100% viable cells.

Following 24 h of exposure, all of the compounds induced a loss of viability, i.e., a certain degree of cytotoxicity towards SH-SY5Y, as shown in Figure 2. Since buprenorphine had the most noticeable effect on the cells, we calculated its IC_50_, which was 58.88 ± 2.20 μM. The IC_50_ for methadone was estimated to be approx. 100 µM based on the observed approx. 50% reduction (dotted red line) at 100 µM, however, complete inhibition (0% response) was not achieved within the tested concentration range. Higher concentrations could not be evaluated because the required vehicle concentration would have been toxic *per se*. Significant decrease was observed across the entire tested concentration range for morphine, codeine, and methadone. For heroin, all tested concentrations except the lowest significantly reduced viability, whereas for buprenorphine, significant effects were observed only at the two highest concentrations tested.

### 3.2. Parameters of Oxidative Stress

**Figure 3:**
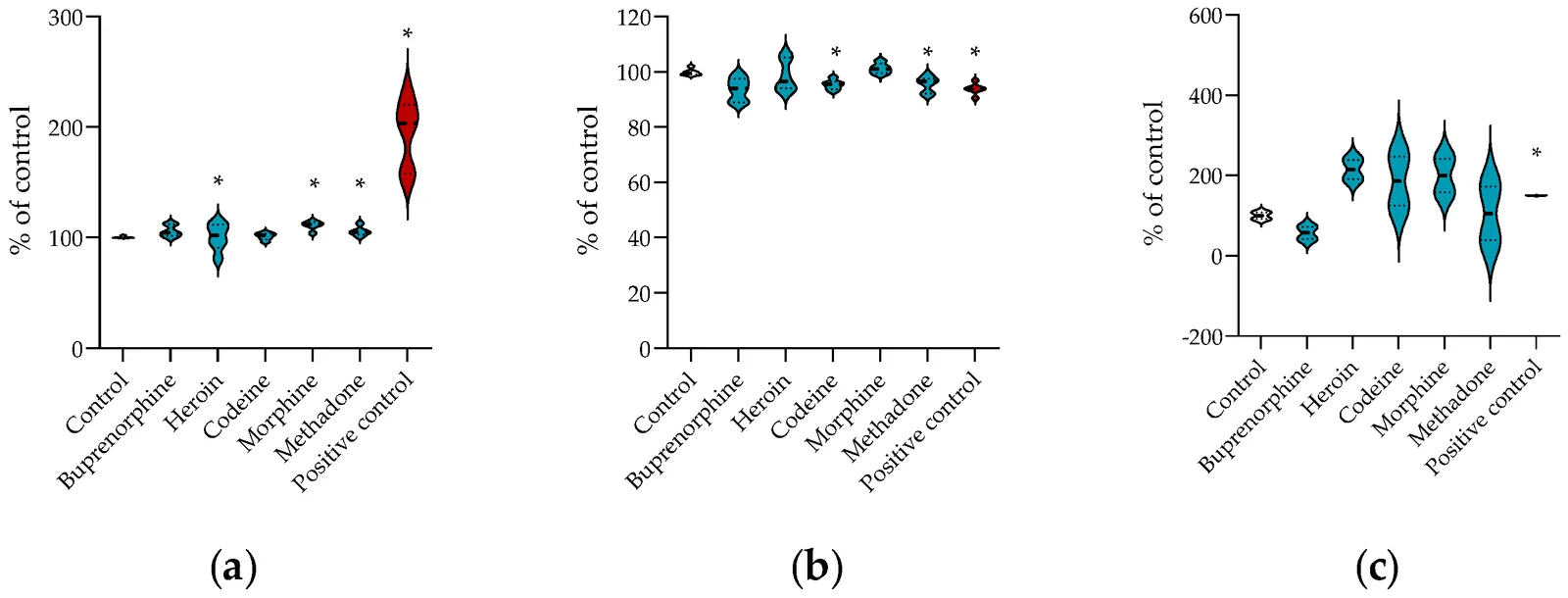
(**a**) Reactive oxygen species (ROS), (**b**) glutathione (GSH), and (**c**) malondialdehyde (MDA) levels in SH-SY5Y cells exposed to opioids (turquoise): buprenorphine (25 μM), heroin (0.156 μM), codeine (1.56 μM), morphine (1.56 μM), and methadone (1.56 μM) for 24 h. Results are expressed as percentage to control untreated cells (Control, white). Positive control (dark red)—H_2_O_2_ treated cells (100 μM). Statistical significance: ANOVA with *post-hoc* Tukey HSD test. * *p* < 0.05 vs. control untreated cells (Control). Legend: Bold dashed line—median, and dotted line—quartiles.

After 24 h treatment, a significant increase in ROS was detected for heroin, morphine and methadone (Figure 3a). The results presented in Figure 3b show that codeine and methadone at the selected concentrations significantly decreased GSH levels compared to the control. Furthermore, MDA levels were not statistically altered in any treatment group compared to control, with a slight increase for most tested compounds and a slight decrease for buprenorphine (Figure 3c).

### 3.3. Antioxidant Enzyme Activity

**Figure 4:**
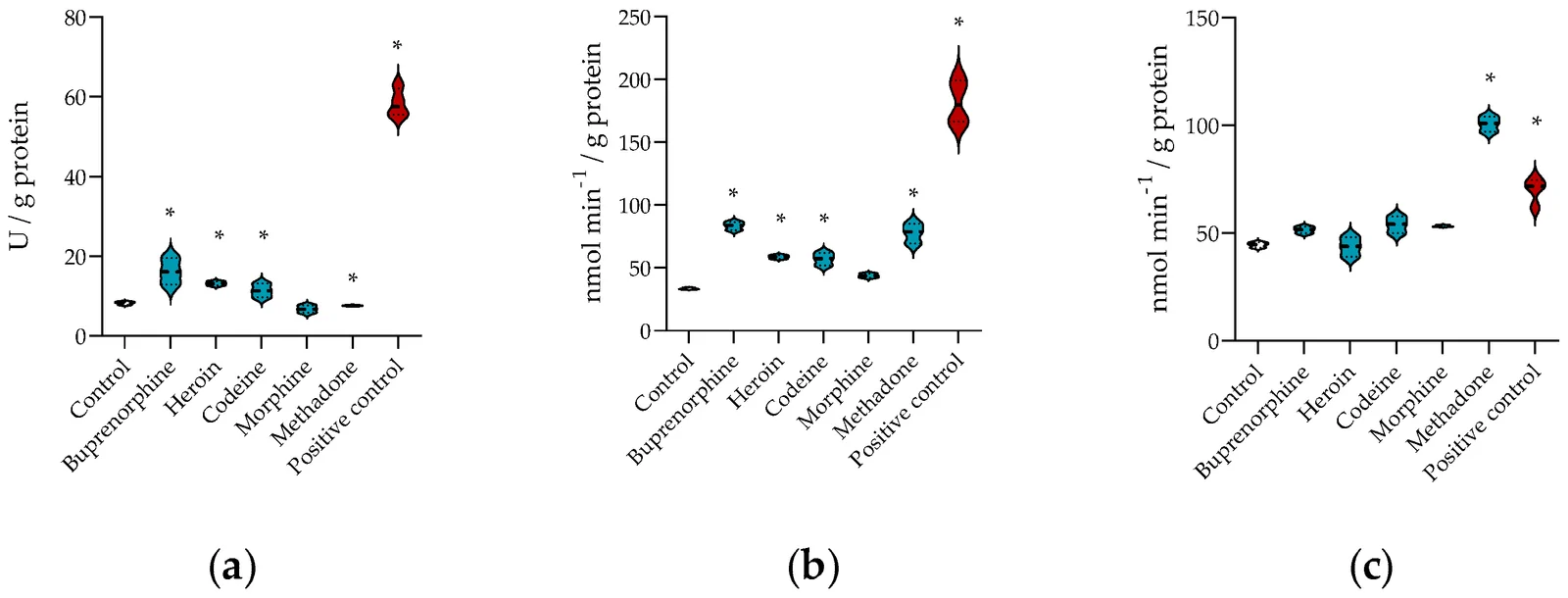
(**a**) Superoxide dismutase (SOD), (**b**) glutathione peroxidase (GPx) and (**c**) catalase (CAT) activity measured in SH-SY5Y cells exposed to opioids (turquoise): buprenorphine (25 μM), heroin (0.156 μM), codeine (1.56 μM), morphine (1.56 μM), and methadone (1.56 μM) for 24 h. Results are expressed as U/g protein (SOD) or nmol/min/g protein (GPx and CAT) to control untreated cells (Control, white). Positive control (dark red)—H_2_O_2_ treated cells (100 μM). Statistical significance: ANOVA with *post-hoc* Tukey HSD test. * *p* < 0.05 vs. control (untreated) cells. Legend: Bold dashed line—median, and dotted line—quartiles.

The results obtained for antioxidant enzyme activities are presented in Figure 4. Compared with untreated controls, cells treated with buprenorphine, heroin, codeine, and methadone showed significantly increased SOD (Figure 4a) and GPx activities (Figure 4b). In contrast, CAT activity was significantly increased only following methadone treatment (Figure 4c). These changes were consistent with the previously observed compound-specific redox responses, particularly the pronounced ROS generation and GSH depletion induced by methadone, whereas the increased antioxidant enzyme activities observed with buprenorphine and heroin occurred in the absence of comparably strong changes in GSH or MDA levels.

### 3.4. Mitochondrial Dysfunction and Membrane Integrity

**Figure 5:**
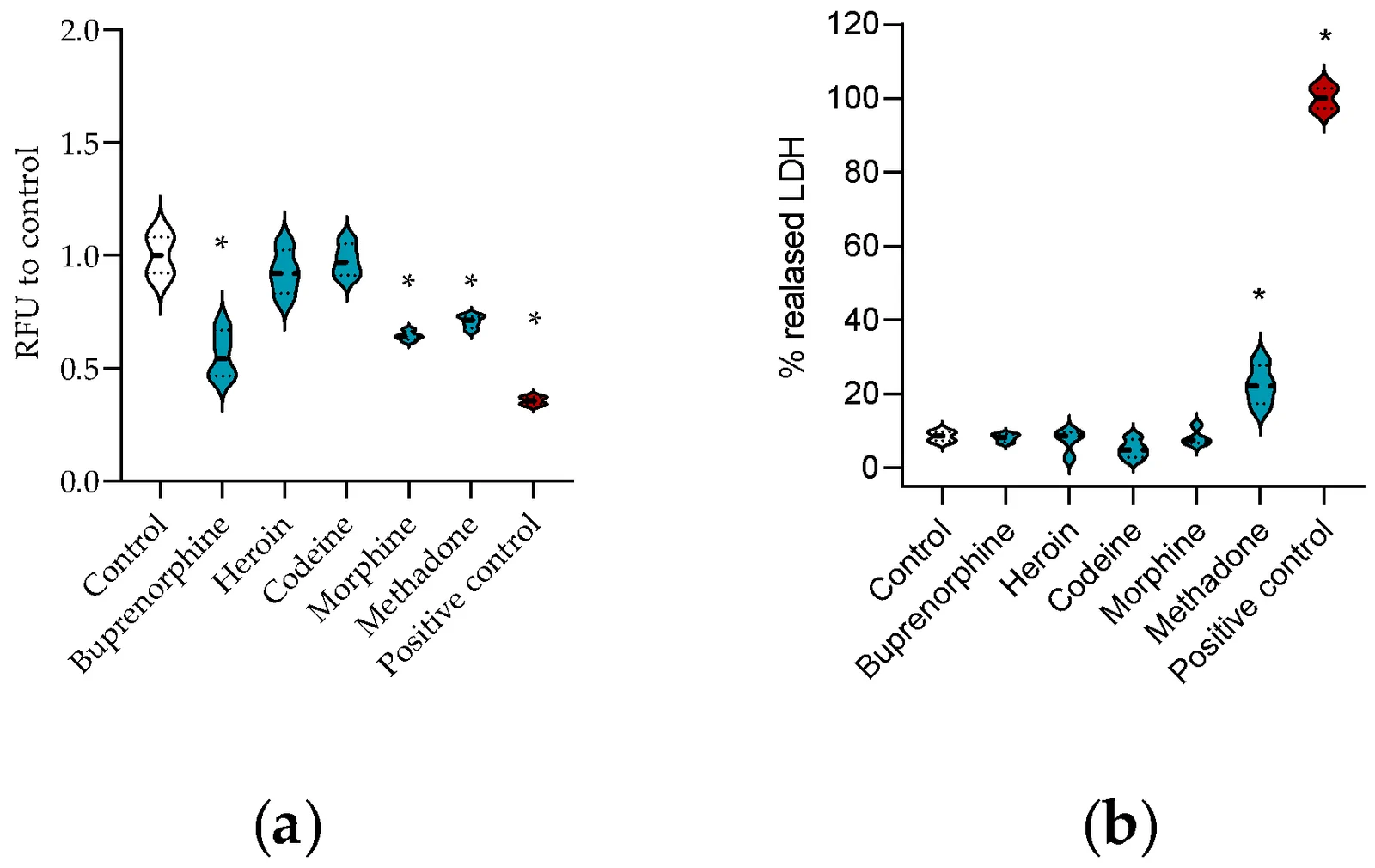
(**a**) Mitochondrial membrane potential (MMP) and (**b**) lactate dehydrogenase (LDH) measured in SH-SY5Y cells exposed to opioids (turquoise): buprenorphine (25 μM), heroin (0.156 μM), codeine (1.56 μM), morphine (1.56 μM), and methadone (1.56 μM) for 24 h. Results are expressed as relative fluorescence units (RFU) to control untreated cells (Control, white) or percentage of LDH release (%) to maximal LDH release (Positive control, dark red). Positive control (dark red)—oxime VII treated cells (100 μM) or Triton X-100 (0.18%, *v*/*v*). Statistical significance: Ordinary one-way ANOVA followed by Dunnett’s multiple-comparisons test. * *p* < 0.05 vs. control (untreated) cells. Legend: Bold dashed line—median; dotted line—quartiles.

Following 24 h exposure, a statistically significant decrease in MMP was observed in cells treated with buprenorphine, morphine, and methadone compared with untreated control cells (Figure 5a). In contrast, a significant increase in LDH release was observed only following methadone treatment, reaching approximately 25% of maximal LDH release, whereas the other tested opioids did not affect LDH release (Figure 5b).

### 3.5. PI3K/Akt Signaling

**Figure 6:**
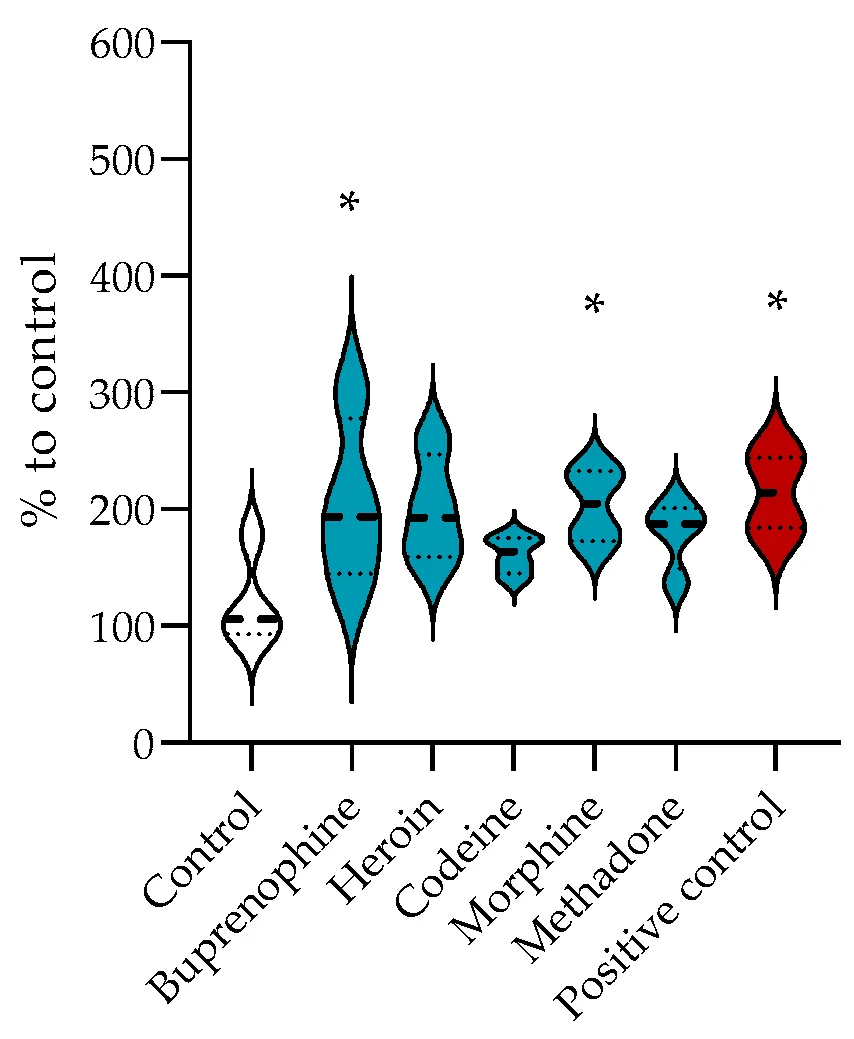
Effects of the tested opioids on PI3K/Akt pathway activation in SH-SY5Y cells exposed to opioids (turquoise): buprenorphine (25 μM), heroin (0.156 μM), codeine (1.56 μM), morphine (1.56 μM), and methadone (1.56 μM) for 1 h. Results are as the percentage of activated Akt-positive cells normalized to untreated control cells (Control, white). Positive control (dark red)—insulin (100 nM) for 1 h. Statistical significance: Ordinary one-way ANOVA followed by Dunnett’s multiple-comparisons test. * *p* < 0.05 vs. control (untreated) cells. Legend: Bold dashed line—median; dotted line—quartiles.

All tested opioids tended to increase Akt activation relative to control cells, suggesting engagement of PI3K/Akt-related survival signaling (Figure 6). This increase reached statistical significance only for buprenorphine and morphine.

### 3.6. Caspase Activation

**Figure 7:**
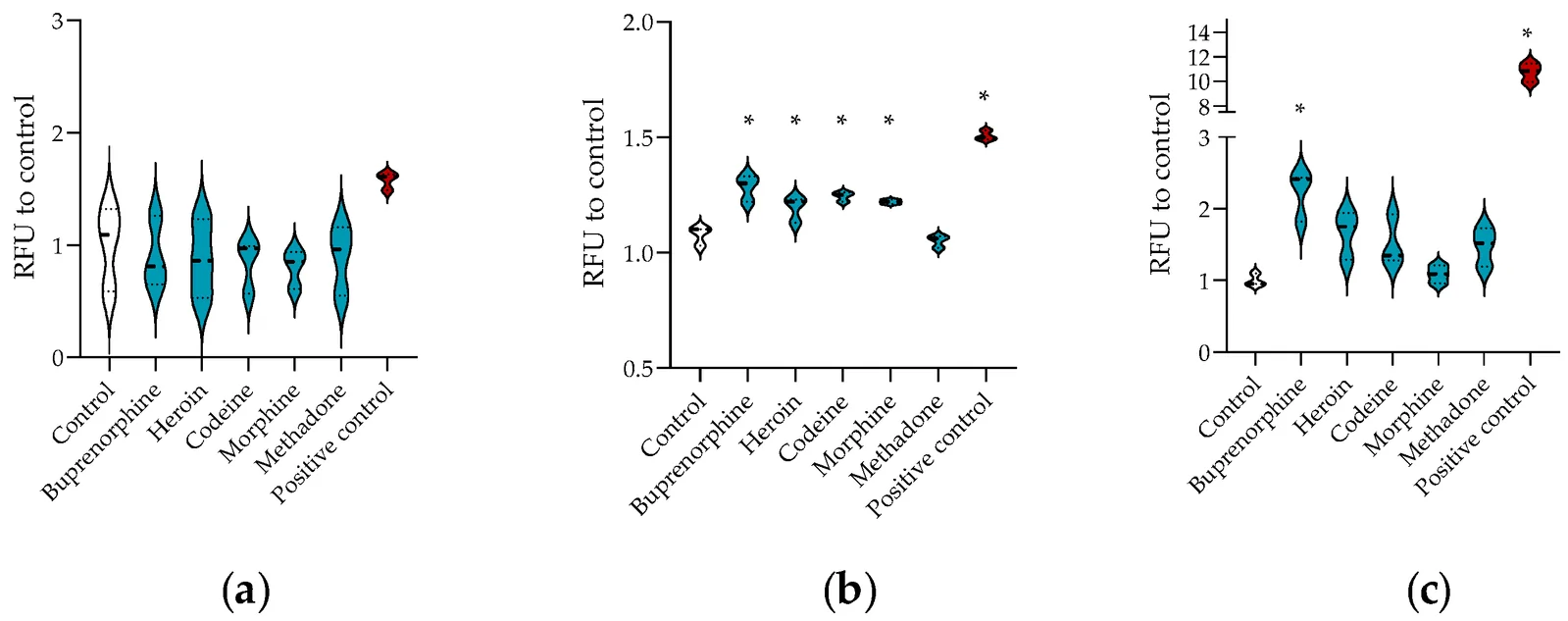
Specific caspase activity: (**a**) caspase 8, (**b**) caspase 9, and (**c**) caspase 3 in SH-SY5Y cells exposed to opioids (turquoise): buprenorphine (25 μM), heroin (0.156 μM), codeine (1.56 μM), morphine (1.56 μM), and methadone (1.56 μM) for 24 h. Results are expressed as relative fluorescence units (RFU) to control untreated cells (Control, white). Positive control (dark red)—staurosporine (SS) treated cells (1 μM). Statistical significance: Ordinary one-way ANOVA followed by a Dunnett’s multiple-comparisons test. * *p* < 0.05 vs. control (untreated) cells. Legend: Bold dashed line—median, and dotted line—quartiles.

After 24 h of opioid exposure, none of the tested compounds significantly affected caspase-8 activity (Figure 7). In contrast, caspase-9 activity was significantly increased by buprenorphine, heroin, codeine, and morphine, whereas methadone produced no significant change despite the observed decrease in mitochondrial membrane potential and pronounced oxidative stress. Caspase-3 activity was significantly increased only by buprenorphine. Heroin and codeine also showed a trend toward increased caspase-3 activity, although these changes did not reach statistical significance. Overall, the results indicate distinct patterns of caspase activation among the tested opioids, with the most pronounced activation observed for buprenorphine.

### 3.7. DNA Strand Breaks

**Figure 8:**
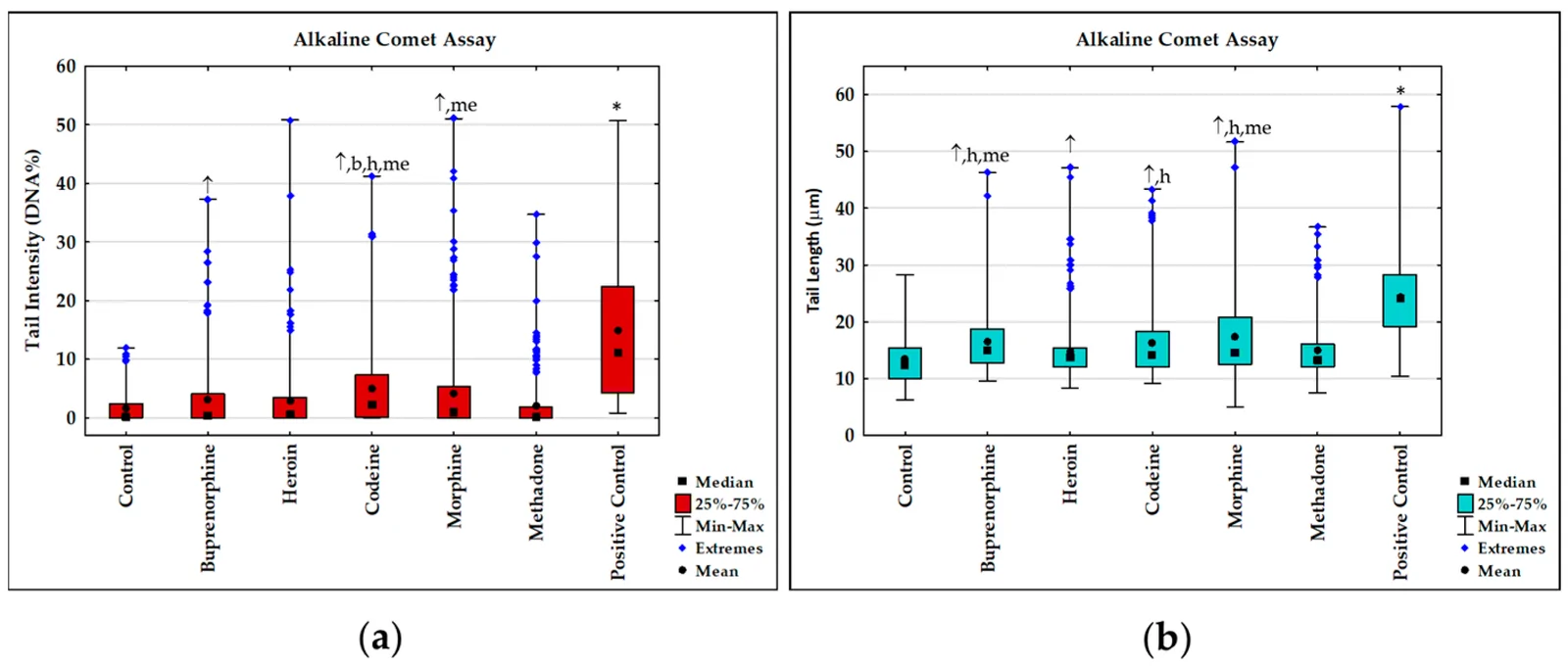
Results of the alkaline comet assay (damage descriptors: (**a**) comet tail intensity and (**b**) tail length) on human neuroblastoma SH-SY5Y cells exposed to buprenorphine (25 μM), heroin (0.156 μM), codeine (1.56 μM), morphine (1.56 μM) and methadone (1.56 μM) for 24 h. DNA strand breaks levels in control (untreated cells) and positive control (hydrogen peroxide 100 μM, for 10 min on ice) were studied in parallel. Descriptive statistics refer to 300 individual comet measurements per each experimental group. Statistical significance was tested using ANOVA with *post-hoc* Tukey HSD test. Significantly increased values (*p* < 0.05) were: **↑** vs. control (untreated cells); **b** vs. buprenorphine; **h** vs. heroin; **me** vs. methadone; * vs. all experimental groups.

Figure 8 summarizes the findings obtained by the alkaline comet assay on SH-SY5Y cells exposed for 24 h to the selected concentrations of tested compounds. Because the method relies on the estimation of DNA strand breaks in single cells, interpretation of the results considered descriptive statistics measures both of central tendency (median, mean) and dispersion (range), determined in the complete dataset for each experimental group. The charts obtained for tail intensity and tail length show specific inter-group differences related to DNA strand breaks levels. The analysis of complete data suggests that at the same molar concentration (1.56 µM), codeine and morphine induced higher levels of DNA strand breaks than methadone. Under these experimental conditions, heroin (at 0.156 µM) caused the lowest level of DNA strand breaks, whereas buprenorphine (at 25 µM) produced only a modest effect. The presence of cells with higher values of comet descriptors (extremes) indicates a greater chance that more complex pathways will have to be used to repair DNA lesions, which in turn may entail a greater likelihood of mutations or severe cytogenetic abnormalities.

### 3.8. HSA Interaction and Cholinesterase Inhibition

The results of HSA binding and cholinesterase inhibition screening are presented in Table 2. Overall, the tested opioids produced only minor quenching of HSA intrinsic fluorescence, ranging from 0.22 to 6.92%, indicating limited interaction with HSA under the applied experimental conditions. The highest fluorescence quenching was observed for buprenorphine. AChE inhibition was relatively low for all tested opioids, with the highest inhibition observed for methadone (20.52%), followed by buprenorphine (19.09%). Similarly, heroin, codeine, morphine, and buprenorphine showed low BChE inhibition, while methadone markedly inhibited BChE activity (79.28%).

### 3.9. In Silico Pharmacokinetic Profiling

**Figure 9:**
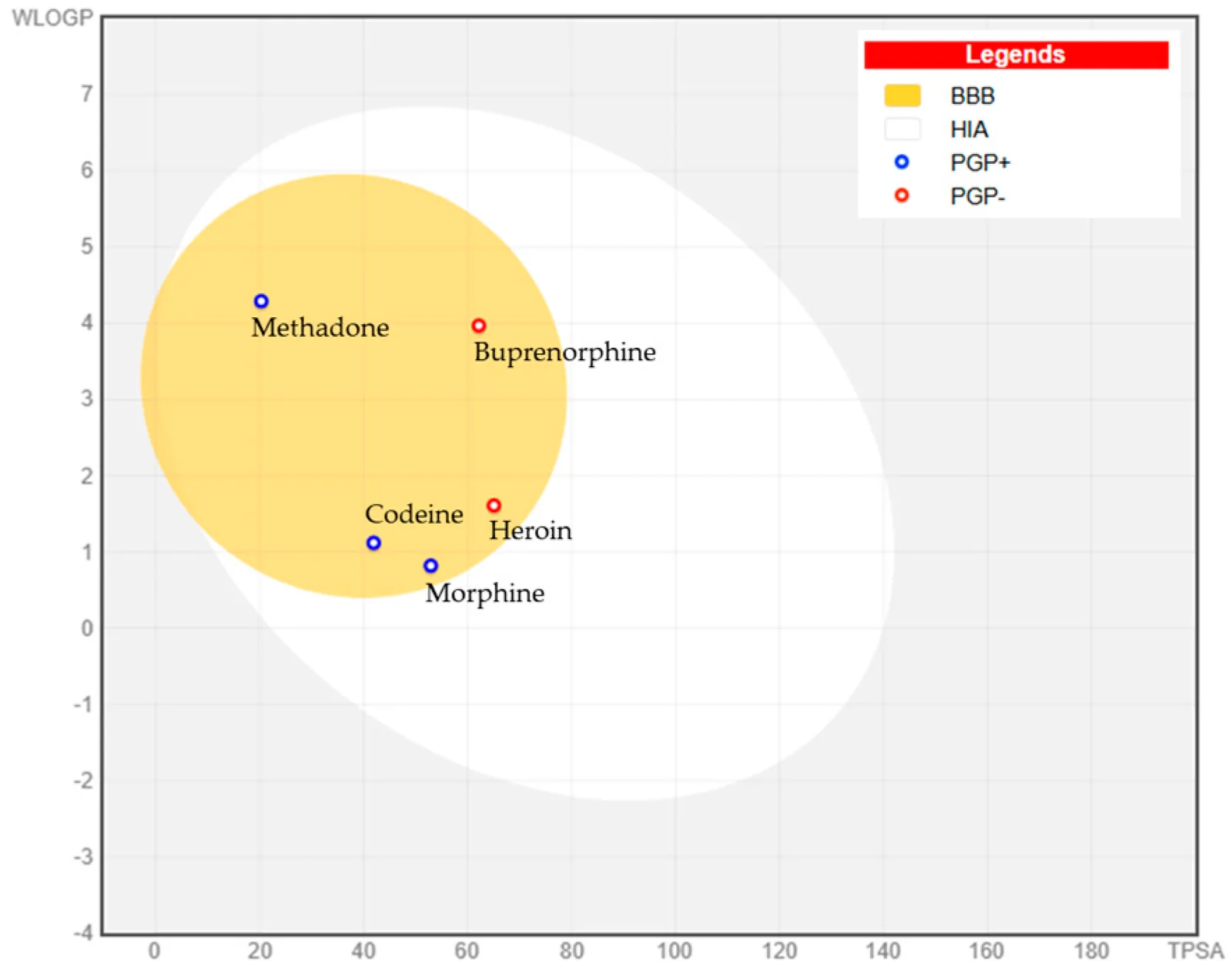
BOILED-Egg prediction of gastrointestinal absorption, blood–brain barrier (BBB) permeation, and P-glycoprotein (P-gp) substrate status of the tested opioids. The white region represents a high probability of passive human intestinal absorption (HIA), whereas the yellow region (yolk) indicates a high probability of BBB permeation. Blue circles indicate compounds predicted to be P-gp substrates (P-gp+), while red circles indicate predicted non-substrates (P-gp−). WLOGP, lipophilicity; TPSA, topological polar surface area. Predictions were generated using SwissADME platform [21].

The BOILED-Egg analysis predicted high human intestinal absorption (HIA) and BBB permeation for all tested opioids (Figure 9). The compounds occupied different positions within the BOILED-Egg plot according to their WLOGP and topological polar surface area (TPSA) values, reflecting differences in lipophilicity and polarity. Methadone and buprenorphine showed the highest WLOGP values, whereas morphine and codeine were positioned at lower lipophilicity, with heroin showing an intermediate profile. Despite these physicochemical differences, all compounds remained within the region associated with high predicted HIA and BBB permeation. Differences were also observed in predicted P-glycoprotein (P-gp) substrate status: morphine, codeine, and methadone were classified as P-gp substrates (P-gp+), whereas heroin and buprenorphine were predicted as non-substrates (P-gp−). Thus, although all opioids showed physicochemical properties compatible with intestinal absorption and central nervous system (CNS) penetration, the predicted P-gp-mediated efflux of morphine, codeine, and methadone may reduce their net brain exposure relative to heroin and buprenorphine.

## 4. Discussion

The principal findings of our study support the initial hypothesis and provide a side-by-side comparison of cytotoxic, redox, mitochondrial, genotoxic, and signaling effects of five structurally distinct opioids in SH-SY5Y cells. By integrating viability, LDH release, oxidative stress markers, mitochondrial membrane potential, caspase activation, PI3K/Akt signaling, DNA strand breaks, HSA interaction, cholinesterase inhibition, and predicted pharmacokinetic properties, the results indicate that opioid-induced cellular toxicity cannot be explained by a single common mechanism. Instead, the tested compounds produced distinct toxicity profiles involving different combinations of oxidative imbalance, mitochondrial dysfunction, apoptotic signaling, membrane damage, and adaptive survival responses.

Consistent with previous in vitro investigations, methadone and buprenorphine showed the highest cytotoxic potential, whereas morphine exerted comparatively limited effects on cell viability [8,10]. These observations agree with studies reporting pronounced cytotoxic and mitochondrial effects of methadone in neuronal models, while morphine often produces milder viability effects under comparable exposure conditions [8,10]. The additional LDH and mitochondrial membrane potential measurements further demonstrated that similar reductions in viability did not necessarily reflect the same type of cellular injury. Buprenorphine, morphine, and methadone significantly decreased mitochondrial membrane potential (MMP), whereas a significant increase in LDH release was observed only for methadone, reaching approximately 25% of maximal release. Thus, mitochondrial dysfunction occurred in the absence of extensive plasma membrane damage for most opioids, while methadone showed a more pronounced membrane-damaging phenotype.

Oxidative stress has frequently been implicated in opioid neurotoxicity, but our findings indicate that its magnitude varies considerably among individual opioids. The CNS is particularly susceptible to oxidative imbalance because of its high oxygen consumption and lipid content and relatively limited antioxidant reserve [43,44]. Opioids have previously been shown to affect ROS generation, glutathione metabolism, and antioxidant enzyme activities [45,46,47]. In the present study, heroin increased ROS levels, together with SOD and GPx activities, suggesting activation of an antioxidant response. Methadone produced the most pronounced oxidative profile, characterized by elevated ROS, GSH depletion, and increased CAT and GPx activities. These findings are consistent with previous reports linking methadone exposure to oxidative and mitochondrial stress [48,49]. Buprenorphine produced a different pattern. Despite being the most cytotoxic compound, it caused only limited changes in the measured oxidative stress parameters, while SOD and GPx activities were increased. This dissociation between cytotoxicity and global oxidative stress suggests that ROS generation alone does not account for buprenorphine-induced cellular injury. Morphine similarly produced only modest redox changes, whereas codeine was characterized by GSH depletion and altered antioxidant enzyme activities. Taken together, these data indicate that the tested opioids disturb redox homeostasis through different patterns of pro-oxidant and compensatory antioxidant responses rather than through a uniform oxidative mechanism.

The mitochondrial and caspase results provide further insight into these compound-specific responses. None of the opioids significantly increased caspase-8 activity, indicating that the canonical extrinsic apoptotic pathway was not prominently activated under the present conditions. In contrast, caspase-9 activity was significantly increased by buprenorphine, heroin, codeine, and morphine, suggesting preferential engagement of pathways associated with intrinsic apoptosis. Buprenorphine showed the clearest mitochondrial apoptotic phenotype. It significantly reduced MMP and activated both caspase-9 and caspase-3, while caspase-8 remained unchanged. Together with its pronounced effect on cell viability, this pattern is consistent with mitochondria-associated, caspase-dependent apoptotic signaling. Morphine also significantly reduced MMP and activated caspase-9, although caspase-3 activation did not reach statistical significance. The present findings therefore support initiation of mitochondrial apoptotic signaling by morphine without demonstrating complete downstream execution of apoptosis at 24 h. This interpretation is consistent with previous SH-SY5Y studies showing ROS-dependent mitochondrial dysfunction, cytochrome c release, and activation of caspase-9 and caspase-3 following morphine exposure [18]. Heroin and codeine significantly increased caspase-9 activity without significantly decreasing MMP at 24 h. Both compounds also showed a trend toward increased caspase-3 activity. These findings may indicate partial or temporally distinct activation of intrinsic apoptotic signaling. Because mitochondrial membrane potential was measured only after 24 h, transient earlier depolarization cannot be excluded. Accordingly, the caspase-9 response should not be interpreted as evidence that heroin and codeine follow the same mitochondrial sequence observed for buprenorphine.

Methadone displayed the most clearly divergent pattern. Despite producing marked ROS accumulation, GSH depletion, significant mitochondrial depolarization, and the only significant increase in LDH release, methadone did not significantly activate caspase-3, -8, or -9. This combination indicates that methadone-induced injury under the present conditions is unlikely to be dominated by classical caspase-dependent apoptosis. Previous studies in SH-SY5Y cells similarly demonstrated methadone-induced mitochondrial dysfunction, ATP depletion, and caspase-independent, necrotic-like cell death [19], with apoptosis-inducing factor also implicated in this response [50]. Our findings therefore support a predominantly caspase-independent mitochondrial injury phenotype for methadone. However, because ATP depletion, AIF translocation, and morphological markers of necrosis were not measured directly, a specific necrotic mechanism cannot be conclusively established.

The PI3K/Akt results add an additional signaling dimension to these observations. All opioids showed a tendency toward increased Akt activation, although statistical significance was reached only for buprenorphine and morphine. PI3K/Akt is an important prosurvival pathway and can be activated downstream of μ-opioid receptor signaling. In differentiated SH-SY5Y cells, μ-opioid receptor stimulation has been shown to promote Akt phosphorylation and cell survival through PI3K-dependent mechanisms [20]. The increased Akt activation observed here may therefore represent an adaptive or compensatory response to opioid-induced stress. This interpretation is particularly relevant for buprenorphine and morphine. In both cases, Akt activation occurred together with mitochondrial dysfunction and caspase-9 activation, suggesting that activation of a prosurvival pathway was insufficient to prevent progression toward mitochondrial injury. For buprenorphine, this response was accompanied by significant caspase-3 activation, whereas morphine showed a more limited downstream apoptotic response. The coexistence of Akt activation and pro-death signaling highlights that activation of a survival-associated pathway should not be interpreted in isolation as evidence of cytoprotection.

The genotoxicity results further demonstrate that oxidative stress and DNA strand breaks were not directly proportional. Morphine and codeine induced greater DNA strand breaks than methadone at equimolar concentrations, whereas heroin produced minimal genotoxic effects under the tested conditions. The pattern of DNA strand breaks observed in our study is consistent with previous reports. Both morphine and codeine have been shown to interact directly with double-stranded DNA, with an electrostatic mode of interaction proposed for codeine and an intercalative mode proposed for morphine [51]. In contrast, methadone was not found to be genotoxic, as demonstrated by comet and γH2AX assays in the study by Kaina et al. [52]. An earlier comet assay study by Xu et al. [53] in mice similarly suggested that heroin does not act as a classical direct DNA-reactive genotoxin, but rather induces DNA damage predominantly through secondary mechanisms, such as oxidative stress. Although the detected DNA strand breaks were biologically modest, statistically significant increases occurred against a low baseline level in untreated cells, consistent with acceptable background values for the alkaline comet assay [54]. Importantly, methadone produced the strongest oxidative phenotype but comparatively less DNA strand breaks and no significant caspase activation. Conversely, morphine and codeine showed greater DNA strand breaks despite less pronounced oxidative stress. These findings suggest that DNA strand breaks may reflect a combination of oxidative injury, apoptotic signaling, and DNA repair activity rather than ROS burden alone. Activation of DNA repair pathways can transiently increase detectable strand breaks [55,56,57], and comet assay measurements therefore represent a dynamic balance between DNA damage induction and repair [30].

The HSA and cholinesterase experiments were included as exploratory assessments of non-canonical molecular interactions. HSA fluorescence quenching was low for all tested opioids, ranging from 0.22% to 6.92%. These values should not be interpreted as evidence of negligible plasma protein binding, because the assay detects only interactions that alter the microenvironment of the intrinsic HSA fluorophore and was performed at a single selected opioid concentration. Previous experimental studies have demonstrated binding of opioids, including morphine and methadone, to human serum proteins and albumin [58,59,60,61,62]. Thus, the present results indicate weak HSA fluorescence responses under the applied conditions rather than absence of binding. Cholinesterase inhibition was generally limited, particularly for BChE inhibition by heroin, codeine, morphine, and buprenorphine. Methadone was a clear exception, producing approximately 79% BChE inhibition compared with approximately 21% AChE inhibition. Earlier structure–activity studies have reported marked differences in cholinesterase inhibition among opioid structural classes and identified methadone as a BChE inhibitor [63]. This result therefore suggests an additional off-target interaction that distinguishes methadone from the other opioids.

Finally, BOILED-Egg analysis provided a complementary pharmacokinetic context. All five opioids were predicted to show high human intestinal absorption and BBB permeation despite differences in WLOGP and TPSA. Methadone and buprenorphine showed the highest lipophilicity, heroin an intermediate profile, and morphine and codeine lower lipophilicity. The compounds also differed in predicted P-glycoprotein status: morphine, codeine, and methadone were classified as P-gp substrates, whereas heroin and buprenorphine were predicted as non-substrates. As P-gp-mediated efflux can limit net CNS exposure, these differences may contribute to variation in brain disposition in vivo. Experimental studies have confirmed a role for P-gp in restricting morphine brain penetration [64].

Several limitations of the present study should be acknowledged. The opioids were tested at viability-based, non-equimolar concentrations, which enabled comparison of cellular responses under sublethal conditions but precludes direct comparison of relative toxic potency. In addition, the experiments were performed in a single cell line and primarily at one exposure duration (24 h), and apoptosis/necrosis was inferred from a limited set of functional markers rather than confirmed by multiple orthogonal approaches. For the comet assay, analysis of multiple cells originating from the same experimental replicate may introduce a degree of pseudoreplication if individual cells are treated as fully independent observations. The HSA-binding and cholinesterase assays were intended as exploratory screening experiments and therefore do not provide quantitative binding constants or inhibitory potency values. Finally, the BOILED-Egg pharmacokinetic analysis is computational and should be interpreted as supportive predictive information rather than experimental evidence of absorption, BBB transport, or P-gp-mediated disposition. These limitations should be considered when interpreting the mechanistic and translational implications of the findings.

Overall, the dataset demonstrates that structurally diverse opioids can converge on cellular toxicity through different combinations of redox imbalance, mitochondrial dysfunction, survival signaling, apoptotic activation, membrane damage, and genotoxicity. Buprenorphine showed a pattern consistent with mitochondrial, caspase-dependent injury, while methadone exhibited pronounced oxidative and mitochondrial stress with membrane damage but without significant caspase activation, supporting a predominantly caspase-independent phenotype. Morphine, heroin, and codeine displayed intermediate or distinct patterns of mitochondrial and apoptotic signaling. These findings show that neither ROS generation nor loss of viability alone is sufficient to characterize opioid neurotoxicity and support the value of a multiparametric, compound-specific approach to understanding opioid-induced cellular injury.

## 5. Conclusions

This study demonstrates that structurally diverse opioids induce distinct cellular toxicity profiles in SH-SY5Y cells rather than acting through a single common mechanism. Buprenorphine showed a predominantly mitochondrial, caspase-dependent apoptotic profile, whereas methadone caused pronounced oxidative and mitochondrial stress with membrane damage but without significant caspase activation, consistent with a more caspase-independent mode of injury. Morphine, heroin, and codeine displayed intermediate and compound-specific patterns of redox imbalance, mitochondrial dysfunction, apoptotic signaling, and DNA strand breaks. Overall, the findings show that oxidative stress alone does not adequately explain opioid neurotoxicity and supports the use of a multiparametric approach to characterize compound-specific toxic mechanisms.

## Figures and Tables

**Table 1:** Compound concentrations for assessing oxidative stress and DNA strand breaks, determined based on 24-h SH-SY5Y cell viability.

Compound	Selected Concentration for Detailed Analysis (µM)	Viability of SH-SY5Y at the Used Concentration (%) ± SEM
Buprenorphine	25	95.08 ± 2.66
Heroin	0.156	82.59 ± 2.99
Codeine	1.56	85.82 ± 3.85
Morphine	1.56	80.84 ± 4.14
Methadone	1.56	79.59 ± 2.93

SEM–standard error of the mean (n = 4).

**Table 2:** Percentage of HSA fluorescence quenching and AChE/BChE inhibition by the tested opioids at selected compound concentrations (n = 4). Cholinesterase inhibition was evaluated over a range of acetylthiocholine (ATCh) concentrations (0.1–0.5 mM), and only the highest inhibition observed within this substrate concentration range is presented.

Compound	Compound Concentration (μM)	HSA Fluorescence Quenching (%)	AChE Inhibition (%)	BChE Inhibition (%)
Buprenorphine	25	6.92	19.09	11.15
Heroin	0.16	0.22	14.75	0.40
Codeine	1.56	2.89	15.76	3.54
Morphine	1.56	2.32	12.37	4.42
Methadone	1.56	0.51	20.52	79.28
BW284C51 ^a^	0.018 and 48	/	50.00	50.00
Ethopropazine ^b^	73 and 0.046	/	50.00	50.00
Warfarin ^c^	3.43	50.00	/	/
Ibuprofen ^c^	2.70	50.00	/	/

BW284C51 and ethopropazine were used as positive controls for AChE and BChE inhibition, respectively, whereas warfarin and ibuprofen were used as reference ligands for HSA binding. Reported IC_50_ values are provided for the cholinesterase inhibitors (^a^ [40] and ^b^ [41]), and *K*_d_ values for the HSA-binding reference compounds (^c^ [42]).

## Data Availability

The original contributions presented in this study are included in the article. Further inquiries can be directed to the corresponding author.

## Abbreviations

The following abbreviations are used in this manuscript:

AChE	Acetylcholinesterase
Akt	Protein kinase B
ATCh	Acetylthiocholine
BBB	Blood–brain barrier
BChE	Butyrylcholinesterase
CAT	Catalase
DCFH-DA	2′,7′-dichlorodihydrofluorescein diacetate
DTNB	5,5-dithio-bis-(2-nitrobenzoic acid)
ECACC	European Collection of Authenticated Cell Cultures
GPx	Glutathione peroxidase
GSH	Glutathione
HSA	Human serum albumin
IC_50_	Half maximal inhibitory concentration
LDH	Lactate dehydrogenase
mBCl	Monochlorobimane
MDA	Malondialdehyde
MMP	Mitochondrial membrane potential
MTS	3-(4,5-dimethylthiazol-2-yl)-5-(3-carboxymethoxyphenyl)-2-(4-sulfophenyl)-2H-tetrazolium salt
PBS	Phosphate-buffered saline
P-gp	P-glycoprotein
PI3K	Phosphoinositide 3-kinase
ROS	Reactive oxygen species
SOD	Superoxide dismutase
TMRE	Tetramethylrhodamine ethyl ester perchlorate
TPSA	Topological polar surface area
WLOGP	Wildman–Crippen lipophilicity coefficient
γH2Ax	Gamma-phosphorylated H2A histone family member X
